# Implementation of a Postfracture Care Program in a Private Hospital in Colombia

**DOI:** 10.1155/2020/8208397

**Published:** 2020-09-18

**Authors:** M. A. Sánchez, J. E. Segura, G. Alajmo, J. M Nossa, A. Correa, E. Leal, A. Moscoso, G. A. Pineda, A. C. Aya

**Affiliations:** Clinica del Country, Bogota, Colombia

## Abstract

**Purpose:**

To describe the implementation of a postfracture care program in a private hospital in Colombia, the results achieved after the program's first year, and the challenges encountered.

**Methods:**

A cross-sectional descriptive study of the first year's outcomes. The program was implemented following best practices described in the “Capture the Fracture” framework. We assessed the management of fractures before the launch of the program. A multidisciplinary group was established to collaborate on the diagnosis and treatment of patients with osteoporotic fractures. A full-time program coordinator was appointed. We analyzed the program's clinical outcomes and limitations.

**Results:**

One-hundred and ninety patients were included in the study, with an average age of 76.7. Hip fracture was the most frequent one (33.6%). After the first year of implementing the program, 39.4% of patients received osteoporosis treatment, with an adherence rate of 73%. The incidence of subsequent falls was 5.8% and 1% for new fractures.

**Conclusions:**

The implementation of a program for patients' care with fragility fractures is challenging for healthcare institutions. The role of a full-time coordinator is critical for the proper operation of such programs.

## 1. Introduction

The World Health Organization has designated 2020–2030 as “the Decade of Healthy Aging,” particularly focusing on fragility fractures as a condition with a strong impact on elderly patients and the Global Healthcare System [1]. In the year 2000, more than 9 million cases of fragility fractures were reported, and over 50 million people worldwide were suffering from long-term physical damages due to previous fractures [2]. In 2017, the average cost of care of a hip fragility fracture was over USD 10,000, while the patient's annual cost exceeded USD 40,000 [3]. More than 840,000 osteoporosis-related fractures were expected to occur in 2018 in Latin America alone, with an estimated cost of over 6.5 billion dollars over 5 years [4]. In Colombia, it was estimated that, in 2012, there were 2,609,858 and 1,423,559 women with osteopenia and osteoporosis, respectively. By 2050, these patients could increase to 3,852,000 and 2,101,000, respectively [5]. Hence, a multidisciplinary approach that incorporates primary and secondary prevention measures is key to reduce costs and optimize care.

Over the past 50 years, orthogeriatric programs have struggled to reduce osteoporotic fractures' morbidity and mortality. The Clínica del Country Hospital is a high complexity private institution in Bogota that attends 12.000 emergencies per month, started in 2013, a multidisciplinary program for managing hip fractures [6]. This program identified existing flaws in both the treatment of postfracture osteoporosis and the prevention of refractures. We believed in the importance of establishing a postfracture care program, given the increasing life expectancy in Colombia (74 years in 2019 [7]), the direct relation between age and fragility fractures, and the rate of elderly patients admitted in the emergency room (23%). This program focuses on patient identification, education, treatment, and outpatient follow-up, with the goal of preventing new fractures. It was implemented following the guidelines of the “Capture the Fracture”, an initiative developed by the International Osteoporosis Foundation through its Fracture Liaison Service (FLS) [8]. Evidence shows that FLS programs reduce mortality and the risk of refracture and increase the treatment rate and bone mineral density [9].

There is limited evidence on the effectiveness of FLS programs in private health institutions in Colombia. We investigated the feasibility of these types of programs in our setting.

We aimed to describe the implementation of a postfracture care program for patients with fragility fractures in a private hospital and to analyze the first year's challenges and results.

## 2. Methods

### 2.1. Preliminary Assessment of Fragility Fracture Care

For assessing the management of the fragility fractures in our institution, we analyzed the data from the electronic medical records of 244 patients treated between June 2017 and March 2018. Strikingly, 97% of patients did not receive adequate pharmacological treatment after discharge. Only 14% of the patients were assessed by the Physical Medicine and Rehabilitation Department (Physiatry Department), and this was the only refracture prevention measure taken. These results align with the data from a previous study in our institution, analyzing hip fracture patients' care [6]. The identification of these deficiencies served to design strategies for the development of the program.

### 2.2. Assembling a Multidisciplinary Team

A multidisciplinary team was formed, including healthcare workers from the following departments: Emergency, Family Medicine, Orthopedics, Geriatrics, Internal Medicine, Rehabilitation (Physiatry), and Endocrinology. Three coordinators led the team: administrative coordinator (Deputy Director of Education and Research), medical coordinator (Orthopedic surgeon), and program coordinator (Head Nurse). Other supporting services included Radiology, Physical Therapy, Nursing, Nutrition, and the Pharmacy Department.

Additionally, an orthopedic specialist who was listed in most of the insurance companies was appointed to conduct outpatient follow-up, a task which was carried out in collaboration with the program coordinator. They evaluated clinical outcomes, investigated secondary causes, educated patients, and monitored drop-out and adherence to treatment rates.

## 3. Program Coordination

The responsibilities of the full-time program nurse coordinator included the following: the active search of participants, in-hospital assessment of patients (with prior consent), program dissemination, and secondary prevention education (physical activity, diet, and fall prevention). Other duties included accompaniment during outpatient medical visits, regular follow-up via telephone calls, and database updating and management. In addition, the coordinator was the focal point for the different participating areas—including insurance companies—thus playing a vital role in the program operation.

### 3.1. Alignment with Local Guidelines

Our program protocol followed the guidelines from the II Colombian Consensus for the treatment of postmenopausal osteoporosis [5], allowing us to standardize the comprehensive treatment of patients with fragility fractures, in terms of diagnosis, complementary testing, pharmacological treatment, secondary prevention, and follow-up.

### 3.2. Program Resources and Funding

Estimating the resources needed for the program was crucial for its successful implementation. Funding was required for fragility fracture care protocol and treatment guide design, staff recruitment, regular meetings, and drafting of final report and results analysis. We received support from a biotechnology company, thanks to a cooperation agreement between AMGEN and our institution.

### 3.3. Program Setup and Diffusion

Once the program was established, an opening meeting was organized. The conference aimed to communicate the program's details, namely, operation protocol, participants' roles, identification of patients, and standard comprehensive treatment for patients with fragility fractures.

The Fracture Liaison Service (FLS) program was implemented at our hospital in December 2018; it was developed following with the “Capture the Fracture” Best Practice Framework (BPF) for the secondary prevention of osteoporotic fractures [10, 11].

Follow-up meetings were scheduled every week initially, to streamline the program's processes and to present partial results. In the latter phase, meetings were scheduled monthly.

### 3.4. Study Period and Participants

We used three methods to select patients. First, professionals from all departments involved identified patients with an osteoporotic fracture and notified the program coordinator through phone calls or web messages. Second, the identification of the patients by the program coordinator was performed through an active search guided by reports of the ICD 10 codes listed in Table 1, from the statistics department. This search was carried out weekly, among hospitalized and the emergency room patients. Third, a periodic assessment was conducted on hospitalized patients with a fracture diagnosis scheduled for surgery.

The inclusion criteria were patients over 60 years with a fragility fracture of the distal radius, proximal humerus, hip, or spine, who had been admitted to our hospital's emergency room between December 1, 2018, and November 30, 2019. Exclusion criteria were patients with either pathological fractures or atypical femoral fractures due to the continued use of bisphosphonates and patients who declined enrollment and follow-up.

### 3.5. Data Collection and Analysis

A database was developed to monitor patients and consolidate data. The collected data comprised all aspects relevant to the optimal care standards and facilitated comparisons with the results of other national and global programs.

The database covered 92 variables, including demographics, type of current fracture, past medical history (risk factors for osteoporosis and fractures), type of treatment (medical or surgical), and financial and clinical outcomes (readmissions, reinterventions, morbidity and mortality, fracture-to-treatment interval, and length of hospital stay). A list of all the variables analyzed is available as supplementary material. The data were collected using Excel 365, and analyzed with IBM SPSS v.26 Statistics software.

## 4. Results

**Figure 1:**
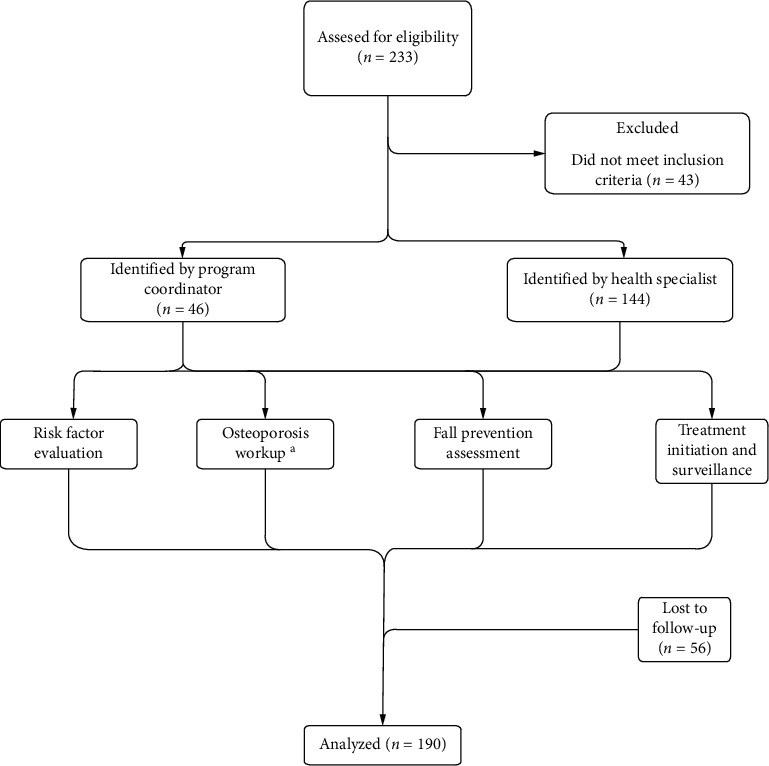
Postfracture care program and study flowchart. ^a^Workup included laboratory testing and dual X-ray absorptiometry (DXA).

The study flow chart of patients is shown in Figure 1. Their treating specialists referred most of the patients. Nearly, a third of patients were identified by the program coordinator (Figure 1).

Most fractures occurred in women (85.8%), and almost half of the study participants were over 80 years old (*n* = 84, mean age 76.7). The majority of patients, 95.2%, presented one or more comorbidities. The most frequent were high blood pressure, hypothyroidism, and type 2 diabetes (Table 2).

**Figure 2:**
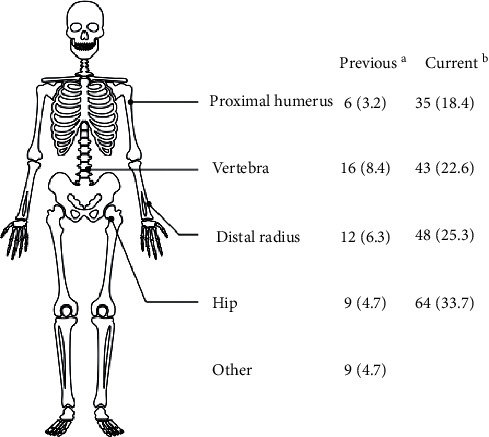
Previous and current fragility fracture sites. Data are expressed as numbers (percentage). ^a^Before the program; ^b^during the study period (year 1 of the program).

Nearly, a fourth of patients (27.3%) had experienced a previous fracture (Figure 2). Although 75% had not been previously diagnosed with osteoporosis, 11% had received antiosteoporotic treatment, and 14% were still taking it at the time of the current fracture. The average time lapse between the previous and the current fracture was 36.7 months.

**Figure 3:**
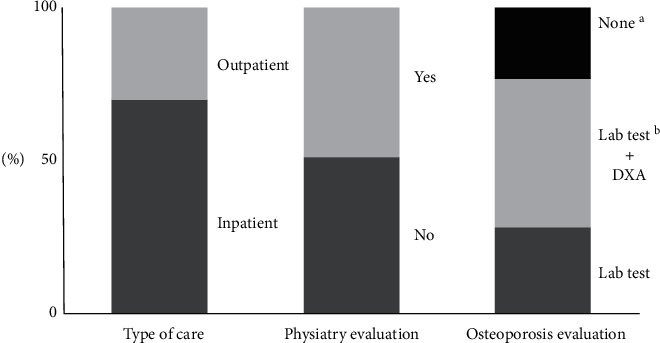
Assessment and care of fragility fracture patients during the study period. ^a^Twenty-three percent of patients (*n* = 44) were not evaluated since they already had a diagnosis of osteoporosis. ^b^Laboratory testing included serum albumin, complete blood count, serum creatinine, vitamin D levels, total calcium, alkaline phosphatase, and parathormone and *DXA* dual-energy X-ray absorptiometry.

A third of the study population received outpatient care, and half of them underwent Physiatry (Rehabilitation Department) evaluation. Twenty-two patients (9.4%) were further evaluated despite already being diagnosed with osteoporosis since they had not completed their previous studies (Figure 3).

While 95% of patients with hip fractures underwent surgery, only 2% of patients with vertebral fractures underwent surgery. More than half of fractured patients required surgery, and these patients were generally operated within 48 hours (Table 3).

During the first year of the program, nearly 40% of patients received treatment for osteoporosis. The most used medications were denosumab and teriparatide (Table 3). The adherence rate to pharmacological therapy during the first year of the program was 73%. By the time of the cut off of the study, 59 patients (31%) completed one year of follow-up, 45 patients (24%) were followed for nine months, and 86 patients (45%) were followed for six months or less.

Once the patients were in the FLS program, new falls and fractures were rare events. One 86-year-old female, firstly enrolled for a right hip osteoporotic fracture treated with a total hip replacement, was readmitted to our hospital two months after being fallen with a right diaphyseal periprosthetic femoral fracture, requiring osteosynthesis. A second readmission case was an 83 yo male, diagnosed with progressive supranuclear paralysis, admitted initially for an L2 osteoporotic fracture treated conservatively, who, three months later, fell from his height during walking, developing vertebral compression fractures in T4, T5, T8, L4, and the left clavicle, treated conservatively again (Table 4). The mortality rate was also low (Table 4). Two patients died during the hospitalization of kidney and respiratory failure. Thirty-day postoperative deaths were due to cardiovascular and respiratory complications.

Deaths occurred a year after surgery because of aortic dissection and bowel obstruction.

## 5. Discussion

We found that fragility fracture patients' outcomes improved after applying the Capture the Fracture Best Practice Framework in our private clinic.

Different multidisciplinary programs for the treatment of fragility fractures, known as postfracture care programs, have been described in the literature. However, a lack of standardization of interventions makes it difficult to establish an ideal model. Fracture Liaison Services (FLSs)—a type of postfracture care program—aim to increase the diagnosis and treatment of low-energy osteoporosis-related fractures and to enhance communication between healthcare providers. FLSs are multidisciplinary services provided by healthcare professionals involved in the care of osteoporosis patients. FLS teams include Orthopedists, Gynecologists, Endocrinologists, Rheumatologists, Physiatrists, Head Nurses, and Nutritionists. Their role is to ensure comprehensive management.

According to the IOF's “Capture the Fracture,” FLS programs are classified into four categories (A, B, C, and D) [12]. Our institution's postfracture care program is a Type A (identify, evaluate, and initiate treatment). It allowed us to identify 190 fragility fracture patients during its first year of implementation, whose demographic characteristics are similar to those reported in the literature.

Remarkably after hip fractures, distal radius fractures (DRFs) were more frequent than vertebral ones, correlating with a reported increase in these fractures in people older than 65 [13]. Otherwise, vertebral fractures remain a diagnosis and treatment concern. Many patients are asymptomatic; others experience mild symptoms, making them look for regular appointments out of the emergency room (ER), delaying the diagnosis; or even worse, some fractures are missed in symptomatic patients during the assessment in the ER because the diagnosis relays in the radiologist inform [14, 15].

In the Colombian context, it is not surprising that nearly two-thirds of patients with a previous fracture had not been diagnosed with osteoporosis, thus confirming that underdiagnosis is a critical problem in our healthcare system [16].

Evidence shows that FLS multidisciplinary models with a central coordinator achieve better results than individual models consisting only of a primary care physician [17]. The central coordinator identifies patients, organizes multidisciplinary teams, engages in treatment, educates patients, and follows every individual.

The Head Nurse played a crucial role in our experience, actively recruiting nearly 24% of patients and promptly identifying underreported vertebral fractures (old or new) helping us encourage the Radiology department to report cases more consistently. As a result, vertebral fractures became the third most frequent one in the study group.

During the first year of our FLS program, 76% of patients were evaluated for osteoporosis (by metabolic profile and bone densitometry), conversely to the findings of a 2005 report from four Colombian hospitals describing that only 10% of patients with hip fractures were assessed for osteoporosis [18]. Following the therapeutic recommendations from the II Colombian Consensus for the Treatment of Postmenopausal Osteoporosis [5], patients with fragility fractures should be treated with denosumab or teriparatide, considering their elevated risk to develop new fractures. In patients having clinical conditions that contraindicate these two medications, zoledronic acid was the option. A previous assessment in 2018 showed a shocking 97% of patients with osteoporotic fractures not adequately treated after discharge in our institution. In opposition, an encouraging finding is that forty percent of the study participants were treated after their enrollment in the FLS program despite the administrative barriers posed by insurance companies.

No less significant, by 2018, in the Clinica del Country hospital, just 14% of patients with osteoporotic fractures had a rehabilitation specialist assessment. At the time of this report, 51% of patients were evaluated by the Rehabilitation Department, receiving therapy and specific guidance to prevent falls. The head coordinator also educated patients and family members on osteoporosis and its treatment. We observed a low rate of complications, reintervention, refracture, and mortality. All deaths were unrelated to the current fragility fracture; they were associated with patients' previous comorbidities.

Osteoporosis undertreatment is a well-established problem worldwide since only 20 to 28% of the osteoporotic diagnosed patients receive treatment [14, 15, 19]. After one year, a high rate of patients untreated for osteoporosis seems to be frustrating. Of course, this index is affected by some patients' inconsistent follow-up; in fact, 29% of the whole group did not continue participating in the FLS program. The main reasons to withdraw were not answering the second phone call (24 patients) and refusing to continue the follow-up process (8 patients). Notwithstanding the high sociocultural status of patients in our institution, seven patients opt out of the program because they were skeptical about the medication or refused to receive a daily injection.

We faced three main obstacles during patient follow-up: some failed to attend their appointments (especially hip fracture patients), and others missed their outpatient complementary studies or did not answer our telephone calls.

Osteoporosis increases the likelihood of fragility fractures [20] in a population already at risk of significant morbidity and premature death due to associated diseases [21]. Aging worsens the impact that fragility fractures have both on the economy and quality of life. Since more than 50% of postmenopausal women and 30% of men over 60 will suffer at least one fragility fracture during their lifetime, this condition has become a significant cause of disability globally [22].

Starting this kind of program implies solving issues about funding and coverage. FLS can raise short-term costs due to an increment in diagnostic tests and pharmacological treatments that stakeholders challenge. Zoledronic acid, denosumab, and teriparatide are expensive drugs, administered for long periods, making barely affordable for patients. The Colombian healthcare system affords the osteoporosis treatment. However, some constraints avoid delivering the treatments for inpatients. The system allows private insurers shift the economic burden to the health-promoting companies who manage the healthcare resources from the Mandatory Health Plan and who prefer to enroll the osteoporotic patients into complex authorization processes that would sometimes last until six months, delaying the treatment or even worse, discouraging them from being treated.

Evidence shows that FLS programs, especially Type A, are efficient. They have demonstrated to increase the diagnosis of osteoporosis—up to 80%—, lower the risk of refracture (30–40%), and reduce both costs and mortality [23, 24]. A meta-analysis calculated that 20 patients would need to be treated to prevent one refracture within three years [24]. The indicators for measuring the impact of our program's implementation were based on those stipulated by the IOF/FFN/NOF [25]. However, given the short time, it has been in operation, and they have not yet revealed the expected impact according to the literature. Cost analysis of the implementation was not in this paper's scope, but we expect the impact on fracture reduction and prevention will make the program very cost-effective in the long term. A cost analysis study is underway.

Our study's main limitation is the short follow-up (12 months) since the mean refracture period reported is approximately three years. Hence, the low incidence of refractures observed (1%) cannot be attributed to the FLS program. Given the nature of our institution, another handicap was raising awareness among the medical staff. We overcame this barrier, thanks to the leadership of the Education and Research Department. They accomplished activities and regular meetings to promote the program, garnering everyone's support and commitment in the process.

Ours is the first report on the implementation of an FLS program in a private hospital in Colombia. After the first year of operation of our program, its clinical outcomes were evaluated. Based on the positive results, the funding parties have agreed to continue providing the program's continuation resources.

We implemented a postfracture care program—in a private practice setting—despite the gaps in insurance coverage and access to treatment. A full-time coordinator is critical for its proper functioning. During the first year of the program, our success was warranted by the coordinator's guidance in connecting the healthcare providers, organizing the database, and measuring the outcomes.

Healthcare institutions worldwide struggle to put in practice the FLS programs. Long-term follow-up of these patients is crucial but challenging—especially in countries such as Colombia. We expect this paper encourage other institutions to start FLS programs.

## Figures and Tables

**Table 1:** ICD 10 codes used for the diagnosis of fragility fractures.

Diagnosis	ICD10 code
Fracture of thoracic vertebra	S220
Fracture of femur, part unspecified	S729
Fracture of lower end of radius	S525
Fracture of upper end of humerus	S422
Unspecified spine fracture	T08X

**Table 2:** Patients' comorbidities and fall risk factors.

Type of disease	*n* = 190
*Cardiovascular or inflammatory disease*	
Hypertension	104 (54.7)
Type 2 diabetes	22 (11.6)
Rheumatoid arthritis	7 (3.7)

*Medication or substance use*	
Antidepressants	18 (9.5)
Alcohol	2 (1.1)
Corticosteroids	6 (3.2)
Smoking	10 (5.3)

*Others*	
Neurological disorder^a^	15 (7.9)
COPD	21 (11.1)
Hypothyroidism	43 (22.6)

*COPD*: chronic obstructive pulmonary disease. Data are expressed as numbers (percentage). ^a^Alzheimer's disease, Parkinson's disease, or dementia.

**Table 3:** Postfracture treatment outcomes.

Outcomes	*n* = 190
*Type of treatment*	
Medical	83 (43.7)
Surgical	107 (56.3)
Radius osteosynthesis	37 (19.5)
Hip osteosynthesis	35 (18.4)
Total hip replacement	23 (12.1)
Total shoulder replacement	4 (2.1)
Humerus osteosynthesis	3 (1.6)
Partial hip replacement	3 (1.6)
Vertebroplasty	2 (1)

*Time to surgery*	
Less than 48 h	94 (87.8)
48 h or more	13 (12.1)

*Hospital stay* (*days*)	
Hospitalization	4.7 ± 4.3
Intensive care unit	2.7 ± 1.4

*Osteoporosis treatment*	
No	117 (61.6)
Yes	73 (39.4)
Denosumab	31 (16.3)
Teriparatide	29 (15.3)
Zoledronic acid	5 (2.6)
Alendronate	5 (2.6)
Ibandronate	3 (1.6)

*Supplementary treatment* ^a^	
Yes	120 (63.1)
No	70 (36.8)

Data are expressed as numbers (percentage) or mean ± standard deviation (SD).^a^ Calcium and vitamin D supplements.

**Table 4:** Complications and morbidity outcomes during the first year of the program.

Outcomes	*n* = 190
Nosocomial complications	4 (2)
Readmission within 30 days	23 (12)
Reintervention	2 (1)
Refracture	2 (1)
Falls after hospital discharge	11 (5.8)

*Mortality*	
During hospitalization	2 (1)
Early (<30 days)	2 (1)
Late (>30 days)	2 (1)

Data are expressed as numbers (percentage).

## Data Availability

The data used to support the findings of this study are available from the corresponding author upon request.

## References

[1] Dreinhöfer K. E., Mitchell P. J., Bégué T. (2018). A global call to action to improve the care of people with fragility fractures. *Injury*.

[2] Johnell O., Kanis J. A. (2006). An estimate of the worldwide prevalence and disability associated with osteoporotic fractures. *Osteoporosis International*.

[3] Williamson S., Landeiro F., McConnell T. (2017). Costs of fragility hip fractures globally: A systematic review and meta-regression analysis. *Osteoporosis International*.

[4] Aziziyeh R., Amin M., Habib M. (2019). A scorecard for osteoporosis in four Latin American countries: Brazil, Mexico, Colombia, and Argentina. *Archives of Osteoporosis*.

[5] Medina Orjuela A., Rosero Olarte Ó., Nel Rueda Plata P. (2018). II Consenso Colombiano para el Manejo de la Osteoporosis Posmenopáusica. *Revista Colombiana de Reumatología*.

[6] Nossa J. M., Escobar N., Márquez D., Leal E., Cabal F., Barreto A. (2016). Aplicación de un programa multidisciplinario para el manejo de fracturas de cadera en el adulto mayor. Incidencia de comorbilidades y su impacto en la oportunidad quirúrgica. *Revista Colombiana de Ortopedia y Traumatología*.

[7] DANE censo general 2005, https://www.dane.gov.co/index.php/estadisticas-por-tema/demografia-y-poblacion/censo-general-2005-1

[8] McLellan A. R., Gallacher S. J., Fraser M., McQuillian C. (2003). The fracture liaison service: success of a program for the evaluation and management of patients with osteoporotic fracture. *Osteoporosis International*.

[9] Wu C.-H., Tu S.-T., Chang Y.-F. (2018). Fracture liaison services improve outcomes of patients with osteoporosis-related fractures: A systematic literature review and meta-analysis. *Bone*.

[10] IOF Fracture Working Group, Åkesson K., Marsh D., Marsh D. (2013). Capture the fracture: A best practice framework and global campaign to break the fragility fracture cycle. *Osteoporosis International*.

[11] IOF capture the fracture® best practice framework for fracture liaison services, 2018, https://www.capturethefracture.org/best-practice-framework

[12] Eisman J. A., Bogoch E. R., Dell R. (2012). Making the first fracture the last fracture: ASBMR task force report on secondary fracture prevention. *Journal of Bone and Mineral Research*.

[13] Ostergaard P. J., Hall M. J., Rozental T. D. (2019). Considerations in the treatment of osteoporotic distal radius fractures in elderly patients. *Current Reviews in Musculoskeletal Medicine*.

[14] Pandya J., Ganda K., Ridley L., Seibel M. J. (2019). Identification of patients with osteoporotic vertebral fractures via simple text search of routine Radiology reports. *Calcified Tissue International*.

[15] Mitchell R. M., Jewell P., Javaid M. K., McKean D., Ostlere S. J. (2017). Reporting of vertebral fragility fractures: Can radiologists help reduce the number of hip fractures?. *Archives of Osteoporosis*.

[16] García J., Guerrero ÉA., Terront A. (2014). Costs of fractures in women with osteoporosis in Colombia. *Acta Medica Colombiana*.

[17] Mitchell P., Åkesson K., Chandran M., Cooper C., Ganda K., Schneider M. (2016). Implementation of models of care for secondary osteoporotic fracture prevention and orthogeriatric models of care for osteoporotic hip fracture. *Best Practice & Research Clinical Rheumatology*.

[18] Londoño R., Soto C., Rueda G., Forero J. H. (2005). La conducta del ortopedista en la prevención, diagnóstico y tratamiento de la osteoporosis en mujeres posmenopáusicas con fractura de cadera en cuatro hospitales de Bogotá-Colombia 2001-2003. *Revista Colombiana de Ortopedia y Traumatología*.

[19] Lorentzon M., Nilsson A. G., Johansson H., Kanis J. A., Mellström D., Sundh D. (2019). Extensive undertreatment of osteoporosis in older Swedish women. *Osteoporosis International*.

[20] National Institute for Health and Care Excellence (2012). *Osteoporosis: Assessing the Risk of Fragility Fracture*.

[21] Bliuc D., Nguyen N. D., Milch V. E., Nguyen T. V., Eisman J. A., Center J. R. (2/4/2009). (AÑO?) mortality risk associated with low-trauma osteoporotic fracture and subsequent fracture in men and women. *JAMA*.

[22] Nguyen N. D., Ahlborg H. G., Center J. R., Eisman J. A., Nguyen T. V. (2007). Residual lifetime risk of fractures in women and men. *Journal of Bone and Mineral Research*.

[23] Sanli I., van Helden S. H., ten Broeke R. H. M. (2019). The role of the Fracture liaison service (FLS) in subsequent fracture prevention in the extreme elderly. *Aging Clinical and Experimental Research*.

[24] Ganda K., Puech M., Chen J. S. (2013). Models of care for the secondary prevention of osteoporotic fractures: A systematic review and meta-analysis. *Osteoporosis International*.

[25] Javaid M. K., Sami A., Lems W. (2020). A patient-level key performance indicator set to measure the effectiveness of fracture liaison services and guide quality improvement: A position paper of the IOF capture the fracture working group, national osteoporosis foundation and fragility fracture network. *Osteoporosis International*.

